# Comprehensive evaluation of primary health care service capacity in China based on TOPSIS and RSR methods

**DOI:** 10.3389/fpubh.2025.1657961

**Published:** 2025-09-23

**Authors:** Lingyi Wang, Furong Li, Shan Jiang, Jingjie Xia

**Affiliations:** Chengdu Center for Disease Control and Prevention (Chengdu Institute of Health Supervision), Chengdu, China

**Keywords:** primary health care, fuzzy comprehensive evaluation, TOPSIS, RSR, entropy weight method

## Abstract

**Background:**

Primary health institutions form the foundation of China’s three-tiered medical and health service system in both urban and rural settings, shouldering the critical role of delivering basic medical and healthcare services to the populace. Gaining insight into the capacity of primary health care services in China post the 2009 New Health Reform is of paramount importance for further safeguarding public health and advancing health equity.

**Methods:**

Using national data from the China Health Statistics Yearbook from 2009 to 2022, we constructed an evaluation system with 14 indicators across three dimensions: health resource input, health service efficiency, and health service output. We employed an entropy-weighted Technique for Order Preference by Similarity to an Ideal Solution (TOPSIS), Rank-Sum Ratio (RSR) method and fuzzy combination evaluation to objectively assess PHC capacity and its constituent parts. Sensitivity analysis was performed to test the robustness of the comprehensive ranking results. The data analysis was meticulously executed using the R4.4.2 software.

**Results:**

The overall comprehensive evaluation score showed a general upward trend, peaking in 2022. However, dimensional analysis revealed a stark divergence: the “Health Resource Input” dimension improved steadily over the period, while the “Health Service Efficiency” dimension showed a clear declining trend, with the most efficient years being at the beginning of the study period.

**Conclusion:**

China’s PHC capacity growth from 2009 to 2022 has been primarily driven by a massive expansion in resources, which masks a persistent and worrying decline in service efficiency. Our objective, multi-dimensional analysis reveals a critical need for a policy pivot from simple resource accumulation to strategic performance optimization. Future efforts must focus on improving the operational efficiency of existing assets and enhancing the quality of care to ensure sustainable and high-value primary health care for the population.

## Background

Primary health care (PHC) serves as the cornerstone of a robust health system (1). As proposed by World Health Organization, primary care is a whole-of-society approach that includes health promotion, disease prevention, treatment and rehabilitation, etc. It addresses the majority of a person’s health needs throughout their lifetime, and it is people-centered rather than disease-centered. Emphasizing primary care is instrumental in ensuring the overall efficiency of the health care system (2). In China, the PHC system provides general clinical care and basic public health services (3). These two components are intricately linked when it comes to disease management and health promotion, highlighting the growing significance of their integration. In 2009, the Chinese government initiated a new wave of health care reforms, identifying four key pillars for the nation’s health system, with equitable and accessible public health services being one of these pillars (4). The National Basic Public Health Service Program offers essential healthcare services aimed at tackling the major health concerns of the population. This program includes both broad population interventions and targeted services for specific groups such as pregnant and postnatal women, children, the older population, and individuals with noncommunicable chronic diseases or tuberculosis, catering to the needs of the entire population across all stages of life (4, 5). In urban areas, the relevant services are delivered by the community health centers (CHCs) or stations, and in rural areas by township health centers (THCs) and village clinics. The stations and clinics provide some services as appropriate under the technical management of CHCs and the THCs, respectively (4). The 2009 health care reform has significantly enhanced accessibility and affordability of PHC through increased government funding, universal health insurance coverage, the implementation of the basic public health service program, and the establishment of an essential drug system (3). The PHC system in China has played a pivotal role in reducing the disease burden. A delivery system centered around PHC would be better aligned with the health needs of the Chinese population (6).

In prior research, the assessment of PHC service capacity predominantly hinged on the annual fluctuations of specific indicators, lacking a coherent evaluation for gaging the overall situation. In this study, we introduce a multifaceted approach employing technique for order preference by similarity to an ideal solution (TOPSIS), rank-sum ratio (RSR) methods and the fuzzy set theory to comprehensively evaluate the dynamic changes of PHC service capacity in China during 2009-2022. This comprehensive evaluation aims to furnish a scientific foundation for enhancing PHC capabilities in China. The TOPSIS method is a technique for determining the order of preference based on the proximity to an ideal solution. It constructs a decision space for both the positive and negative ideal solutions from a normalized raw data matrix. The solutions under scrutiny are treated as spatial points, and the method calculates the distance from each point to the ideal solutions. This metric serves to quantify the relative closeness to the ideal solution, thereby providing a basis for assessing the merits and drawbacks of each solution (7). The TOPSIS method has a wide range of applications and is generally used in evaluation of corporate management, marketing, investment decision-making, health care and other fields (8). The RSR method, introduced by Chinese scholar and professor Tian Fengdiao of the Chinese Academy of Preventive Medicine in 1988 (9), is a classical multi-criteria decision-making approach that has been extensively applied in the comprehensive evaluation of multiple indicators within the medical health sector and beyond (10). Fuzzy set theory has been approved to be an effective approach to deal with uncertainty and ambiguity in multi-criteria decision-making (11). Compared with using the TOPSIS method or RSR alone the fuzzy combination of the two methods is used for comprehensive evaluation, which can make full use of the characteristics and advantages of fuzzy combination, and conduct comprehensive analysis from the perspective of ratio weight and classification calculation method, making the research more distinguishable and measurable (8).

## Data and methods

### Data

The related data were collected from the China Health Statistics Yearbook publicly released by National Health Commission of the People’s Republic of China every year. Population data were derived from the China Statistical Yearbook published annually by the National Bureau of Statistics. The 2009–2022 data for this study was sourced from the Statistics Yearbook 2010–2023. On the basis of previous studies on capacity evaluation of PHC, and following the principles of feasibility and scientific validity, we established an evaluation system comprising three dimensions of health resource input, health service efficiency, and health service output. Table 1 shows the evaluation system and indicators of PHC service capacity. Table 2 shows the original data of PHC service capacity in China from 2009 to 2022.

**Table 1:** Evaluation system of PHC service capacity.

Dimension	Indicator	Indicator type
Health resource input	X1 Number of primary health institutions per 10,000 people	Beneficial
	X2 Number of health professional technicians in primary health institutions per 10,000 people	Beneficial
	X3 Number of beds in primary health institutions per 10,000 people	Beneficial
	X4 The average number of village doctors and health workers in each village clinic	Beneficial
Health service efficiency	X5 The average number of patients per day for CHC physicians	Non-Beneficial
	X6 The average number of patients per day for station physicians	Non-Beneficial
	X7 The average number of patients per day for THC physicians	Non-Beneficial
	X8 Hospital bed utilization rate in CHC	Beneficial
	X9 Hospital bed utilization rate in THC	Beneficial
	X10 Average day of stay hospital in CHC	Non-Beneficial
	X11 Average day of stay hospital in THC	Non-Beneficial
Health service output	X12 Number of patients treated in primary health institutions (Billion person-times)	Beneficial
	X13 Number of patients admitted to hospital in primary health institutions (Ten thousand person-time)	Beneficial
	X14 Number of family health services in primary health institutions (Ten thousand person-time)	Beneficial

**Table 2:** The original data of PHC service capacity in China from 2009 to 2022.

Years	X1	X2	X3	X4	X5	X6	X7	X8	X9	X10	X11	X12	X13	X14
2009	0.49	9.00	7.75	1.66	14	13.7	8.3	59.8	60.7	10.6	4.8	12.54	3971.96	1047.88
2010	0.53	9.73	8.67	1.68	13.6	13.6	8.2	56.1	59	10.4	5.2	13.59	3848.44	1115.28
2011	0.52	10.00	8.99	1.70	14	13.7	8.5	54.4	58.1	10.2	5.6	14.13	3696.12	1244.80
2012	0.52	10.33	9.58	1.67	14.8	14	9.1	55.5	62.1	10.1	5.7	15.66	4176.17	1465.91
2013	0.52	10.60	9.73	1.67	15.7	14.3	9.3	57	62.8	9.8	5.9	16.64	4229.21	1452.49
2014	0.52	10.69	9.90	1.64	16.1	14.4	9.5	55.6	60.5	9.9	6.3	17.14	4030.66	1479.70
2015	0.51	10.91	10.10	1.61	16.3	14.1	9.6	54.7	59.9	9.8	6.4	17.61	3981.61	1466.65
2016	0.51	11.22	10.25	1.57	15.9	14.5	9.5	54.6	60.6	9.7	6.4	18.01	4113.66	1490.95
2017	0.51	11.61	10.79	1.53	16.2	14.1	9.6	54.8	61.3	9.5	6.3	18.78	4391.42	2011.64
2018	0.51	11.96	11.14	1.46	16.1	13.7	9.3	52	59.6	9.9	6.4	19.15	4324.62	2129.58
2019	0.50	12.46	11.40	1.37	16.5	13.9	9.4	49.7	57.5	9.7	6.5	20.34	4248.91	2558.19
2020	0.50	12.93	11.53	1.30	13.9	10.8	8.5	42.8	50.4	10.3	6.6	18.50	3676.07	2656.33
2021	0.50	13.28	11.82	1.15	14.6	11	8.9	43.2	48.2	9.8	6.6	19.97	3542.30	4067.00
2022	0.50	13.81	12.18	1.19	13.9	11	9.1	41.1	46.9	9.9	6.5	20.40	3572.82	12827.94

## Methods

### Entropy weight method

The entropy weight method is a weighting approach grounded in information entropy. It quantifies the amount of useful information contained in each indicator’s data set, subsequently determining its weight in an objective manner without relying on subjective judgment. Using entropy method to determine the index weight of the processed data can make it more objective and comparable (8, 11). The detailed processes are as follows:

1. According to Table 2, we establish the following judgment matrix X Equation 1: $X=(x_{\mathrm{ij}})\;m,n$ (i = 1,2,…,m; j = 1,2,…,n). In which n = 14, m = 14, *x_ij_* represents the value of the *j*-th indicator in the *i*-th year.

(1) $$X=[\begin{array}{l} x_{11}\;\;\;x_{12}\;\;\ldots x_{1n}\\ x_{21}\;\;\;x_{22}\ldots x_{2n}\\ \ldots \;\ldots \;\ldots \;\ldots \\ x_{m1}\;\;\;x_{m2}\ldots x_{\mathrm{mn}} \end{array}]$$

2. Within the entropy weight method, a fundamental distinction exists between beneficial (higher values are preferable) and non-beneficial (lower values are preferable) criteria. We use Equations 2, 3 to normalize beneficial and non-beneficial indicators, respectively.

(2) $$r_{\mathrm{ij}}=\frac{X_{\mathrm{ij}}-(X_{\mathrm{ij}})\min}{(X_{\mathrm{ij}})\max-(X_{\mathrm{ij}})\min}$$

(3) $$r_{\mathrm{ij}}=\frac{(X_{\mathrm{ij}})\max-X_{\mathrm{ij}}}{(X_{\mathrm{ij}})\max-(X_{\mathrm{ij}})\min}$$

3. The information entropy and information were calculated according to the constructed decision matrix Value of utility. The entropy of the *j-*th indicator is the Equation 4. The weight of the *j-*th indicator is the Equation 5.

(4) $$E_{j}=-k\sum_{i=1}^{m}f_{\mathrm{ij}}\ln f_{\mathrm{ij}},f_{\mathrm{ij}}=\frac{r_{\mathrm{ij}}}{\sum_{i=1}^{m}r_{\mathrm{ij}}},k=\frac{1}{\ln m}$$

(5) $$w_{j}=\frac{1-E_{j}}{n-\sum_{j=1}^{n}E_{j}}$$

### Entropy-weighted TOPSIS evaluation method

The basic principle of TOPSIS is to find out the optimal solution and the worst solution in the limited solution based on the normalized original data matrix, and then calculate the distance between each evaluation object and the optimal solution and the worst solution, to obtain the relative proximity between each evaluation object and the optimal solution, as the basis for evaluation (12). The detailed processes are as follows:

1. The TOPSIS method requires that all indicators change in the same trending direction. In the comprehensive evaluation, some are beneficial indicators (such as hospital bed utilization rate, etc.) and some are non-beneficial indicators (such as average day of stay hospital). When using this method for evaluation, the non-beneficial indicators are transformed into beneficial indicators by using reciprocal.

2. The original data matrix after the same trend was normalized and the corresponding matrix was established. The index conversion is Equation 6.


(6) aij=xij∑i=1nxij2

$a_{\mathrm{ij}}$ represents the normalized value of *j*-th indicator’s value in the *i*-th year. Following the entropy weight method, we computed criterion weights *w_j_* and applied them to the normalized values *a_ij_* to construct the weighted matrix A by Equations 7, 8.

(7) $$a_{\mathrm{ij}}=w_{j}\cdot a_{\mathrm{ij}}$$

(8) $$A=[\begin{array}{l} w_{1}a_{11}\;\;w_{2}a_{12}\;\;\ldots w_{n}a_{1m}\\ w_{1}a_{21}\;\;w_{2}a_{22}\ldots w_{n}a_{2m}\\ \ldots \;\;\;\ldots \;\ldots \;\;\ldots \\ w_{1}a_{n1}\;\;w_{2}a_{n2}\ldots w_{n}a_{\mathrm{nm}} \end{array}]$$

3. The optimal value vector and the worst value vector were obtained according to the A matrix, which is the optimal Solution 9 and the worst Solution 10 in the finite scheme, i = 1,2,. m; j = 1,2,., n. $a\prime_{\mathrm{ij}}^{+}$ and $a\prime_{\mathrm{ij}}^{-}$ denote the maximum and minimum values of the existing evaluation objects on the *j-*th indicator, respectively.

(9) $$A\prime +=(a\prime_{i1}^{+},a_{i2}^{\prime +},\ldots ,a_{\mathrm{im}}^{\prime +})$$

(10) $$A\prime -=(a\prime_{i1}^{-},a\prime_{i2}^{-},\ldots ,a\prime_{\mathrm{im}}^{-})$$

4. The distance between each evaluation object from the optimal solution $D_{i}^{+}$ and the worst solution $D_{i}^{-}$ was calculated by Equations 11, 12.

(11) $$D_{i}^{+}=\sqrt{\sum_{j=1}^{m}(a_{\mathrm{ij}}^{\prime +}-a\prime_{\mathrm{ij}})2}$$

(12) $$D_{i}^{-}=\sqrt{\sum_{j=1}^{m}(a_{\mathrm{ij}}^{\prime -}-a\prime_{\mathrm{ij}})2}$$

5. The proximity between each evaluation object and the optimal solution was calculated by Equation 13. The value of *C_i_* is between 0 and 1. When the *C_i_* value is closer to 1, it indicates that primary health services capacity in this year is closer to the optimal level.

(13) $$C_{i}=\frac{D_{i}^{-}}{D_{i}^{+}+D_{i}^{-}}$$

6. To rank each evaluation object, the evaluation effect was determined by calculating the *C_i_* value.

### Entropy-weighted RSR evaluation method

The basic idea of RSR method is a dimensionless statistic RSR obtained by rank transformation in the matrix of n rows and m columns. The concept and method of parameter statistical analysis are used to study the distribution of RSR, and the RSR value is used to directly rank or rank by degree. This method has been widely used in the comprehensive evaluation of multiple indicators in the field of health care (12). The detailed processes are as follows:

1. Organize the evaluation objects into an original data matrix with n rows and m columns. Rank the indicators of PHC, with the beneficial indicators ranked in ascending order and the non-beneficial indicators ranked in descending order. If the value of indicator is the same, the average rank is compiled.

2. The value of weighted-RSR (WRSR) was calculated by Equation 14.

(14) $${\text{WRSR}}_{i}=\frac{1}{\mathrm{mn}}\sum_{j=1}^{m}w_{j}R_{\mathrm{ij}}$$

In the equation, i = 1,2,…,m; j = 1,2,…,n; n = 14, m = 14. *R_ij_* is the rank of primary health capacity indicators in China from 2009 to 2022.

3. Calculate the probability unit (Probit) value. The sorted WRSR values are sorted from small to large (those with the same value are a group). Compile the WRSR frequency distribution table, calculate the frequency (f) and the cumulative frequency of each group, and convert the cumulative frequency into the probability unit value with reference to the “Percentage and Probability Unit Comparison Table” (13).

4. Using the Probit corresponding to the cumulative frequency as the independent variable and WRSR as the dependent variable, the regression equation was presented as Equation 15:

(15) WRSR=a+b×Probit

5. The evaluation objects were ranked by levels based on *WRSR* values.

### The fuzzy combination of TOPSIS and RSR method

The detailed processes are as follows (8, 14):

1. The value of *C_i_* and *WRSR* was calculated through TOPSIS method and RSR method, respectively.

2. The value of *C_i_* and W*RSR* was substituted into the Formula 16.

(16) $$W_{1}C_{i}+W_{2}\times \text{WRSR}$$

The weight ratio *W_1_: W_2_* are taken as 1:0, 0.1:0.9, 0.5:0.5, 0.9:0.1, 0:1, respectively.

3. According to the “most principle” (15), there are n ranks $R_{1},R_{2},\ldots ,R_{n}\in U$ (17) of fuzzy combination of TOPSIS and RSR method and each $R_{i}(i=1,2,\ldots ,n)$ (18) has a feature set expressing a certain attribute. If R_i_ with the same feature set is divided into one group, which can be divided into m group, 1 ≤ m ≤ n, then the feature set containing the most R_i_ in each group is selected, and the feature set is the ranking set of various comprehensive evaluations. Specifically, multiple ranking results derived from different weight combinations were treated as a “feature set.” For each specific rank, the year that most frequently achieved that rank across all evaluations was assigned the corresponding final rank. This approach integrates multiple evaluation outcomes to derive a consensus-based final ordering.

### Sensitivity analysis

The purpose of sensitivity analysis is to identify which parameters have the most significant impact on the model’s results, thereby determining the robustness of the outcomes (11). In the study, sensitivity analysis was conducted by changing weight ratio according to the changing value of *W1* and *W2* of the fuzzy combination of TOPSIS and RSR method. The weight ratio *W1: W2* of sensitivity analysis are applied as 1:0, 0.9:0.1, 0.8:0.2, 0.7:0.3, 0.6:0.4, 0.5:0.5, 0.4:0.6, 0.3:0.7, 0.8:0.2, 0.9:0.1, 0:1, respectively.

### Statistical analysis

Excel 2019 was used for data entry and collation. TOPSIS method and RSR method analysis were performed by R4.4.2, and the test level was *α* = 0.05.

## Results

### The entropy weight for indicators

Table 3 shows each indicator’s entropy weight values, which shows X14 has the maximum weight value of 0.2147, and X10 has the minimum weight value of 0.0342.

**Table 3:** Entropy weight of indicators in evaluation system.

Indicator	X1	X2	X3	X4	X5	X6	X7
Entropy (*E_j_*)	0.9366	0.9240	0.9414	0.9305	0.8725	0.7970	0.8396
Weight (*w_j_*)	0.0381	0.0457	0.0352	0.0418	0.0767	0.1220	0.0964

Indicator	X8	X9	X10	X11	X12	X13	X14
Entropy (*E_j_*)	0.9302	0.9377	0.9431	0.7988	0.9338	0.9082	0.6428
Weight (*w_j_*)	0.0420	0.0374	0.0342	0.1210	0.0398	0.0552	0.2147

### TOPSIS evaluation results

According to the *C_i_* value of TOPSIS method evaluation of PHC overall service capacity in each year, the top 3 from 2009–2022were in 2022, 2021 and 2020, and the last 3 were in 2015, 2011 and 2014. In terms of health resource input, the top 3 years were 2019, 2020, and 2018, whereas the bottom three were 2009, 2010, and 2011. Regarding health service efficiency, the top 3 years were 2009, 2010, and 2011, compared to the bottom three: 2019, 2018, and 2015. As for health service output, the top years were 2022, 2021, and 2020, in contrast to the bottom three: 2010, 2009, and 2011. The results shows in Table 4.

**Table 4:** TOPSIS evaluation of PHC service capacity in China from 2009 to 2022.

Dimension	Health resource input		Health service efficiency		Health service output		Overall service capacity			
Year	Ci	Rank	Ci	Rank	Ci	Rank	Di+	Di-	Ci	Rank
2009	0.3623	14	0.6283	1	0.0164	13	0.1717	0.0137	0.0740	7
2010	0.4321	13	0.6204	2	0.0137	14	0.1707	0.0116	0.0634	8
2011	0.4642	12	0.5699	3	0.0191	12	0.1689	0.0096	0.0536	13
2012	0.5018	11	0.5291	4	0.0399	11	0.1657	0.0105	0.0599	9
2013	0.5260	10	0.4973	6	0.0401	10	0.1660	0.0101	0.0572	10
2014	0.5308	9	0.4424	10	0.0413	8	0.1657	0.0094	0.0537	12
2015	0.5490	8	0.4291	12	0.0408	9	0.1659	0.0092	0.0526	14
2016	0.5663	7	0.4396	11	0.0438	7	0.1656	0.0096	0.0551	11
2017	0.6200	5	0.4511	9	0.0863	6	0.1580	0.0162	0.0930	6
2018	0.6362	3	0.4148	13	0.0958	5	0.1562	0.0177	0.1015	5
2019	0.6393	1	0.3466	14	0.1315	4	0.1501	0.0234	0.1349	4
2020	0.6376	2	0.5282	5	0.1379	3	0.1484	0.0268	0.1527	3
2021	0.5909	6	0.4706	8	0.2572	2	0.1281	0.0456	0.2626	2
2022	0.6323	4	0.4715	7	0.9784	1	0.0129	0.1718	0.9299	1

### RSR sorting and grading results

According to the *WRSR* value ranking in Table 5 of PHC service capacity in each year, the probability distribution *Probit* value is calculated in Table 6. Taking the value of *WRSR* in the table as dependent variables and the *Probit* value as independent variables, the regression equation is calculated as: distribution value of *WRSR* = 0.015 + 0.005 × *Probit* (*F* = 448.047, *p* < 0.001), which shows that the difference between the equations is statistically significant and the fitting level is high. The capacity evaluation of PHC service in 2009–2022 is divided into 3 grades, of which 2022, 2020 and 2021 are good grade, 2015 and 2014 are poor grade, and the remaining 10 years are medium grade in Table 7.

**Table 5:** RSR evaluation of PHC service capacity in China from 2009 to 2022.

Years	R1	R2	R3	R4	R5	R6	R7	R8	R9	R10	R11	R12	R13	R14	*WRSR*	Rank
2009	1	1	1	10	10.5	9.0	13.0	14	11	1.0	14.0	1	6	1	0.0357	11
2010	14	2	2	13	14.0	11.0	14.0	12	6	2.0	13.0	2	5	2	0.0416	4
2011	13	3	3	14	10.5	9.0	11.5	6	5	4.0	12.0	3	4	3	0.0375	9
2012	12	4	4	12	8.0	6.0	8.5	10	13	5.0	11.0	4	10	5	0.0390	6
2013	11	5	5	11	7.0	3.0	6.5	13	14	10.0	10.0	5	11	4	0.0362	10
2014	10	6	6	9	4.5	2.0	3.5	11	9	7.0	8.5	6	8	7	0.0327	13
2015	9	7	7	8	2.0	4.5	1.5	8	8	10.0	6.0	7	7	6	0.0293	14
2016	8	8	8	7	6.0	1.0	3.5	7	10	12.5	6.0	8	9	8	0.0333	12
2017	7	9	9	6	3.0	4.5	1.5	9	12	14.0	8.5	10	14	9	0.0388	7
2018	6	10	10	5	4.5	9.0	6.5	5	7	7.0	6.0	11	13	10	0.0411	5
2019	5	11	11	4	1.0	7.0	5.0	4	4	12.5	3.5	13	12	11	0.0376	8
2020	4	12	12	3	12.5	14.0	11.5	2	3	3.0	1.5	9	3	12	0.0439	2
2021	3	13	13	1	9.0	12.5	10.0	3	2	10.0	1.5	12	1	13	0.0430	3
2022	2	14	14	2	12.5	12.5	8.5	1	1	7.0	3.5	14	2	14	0.0460	1

**Table 6:** Frequency distribution of RSR and Probit of PHC service capacity in China from 2009 to 2022.

Years	*WRSR*	*f*	*Cumulative f*	*R*	R/n*100%	*Probit*
2015	0.0293	1	1	1	7.14	3.5348
2014	0.0327	1	2	2	14.29	3.9324
2016	0.0333	1	3	3	21.43	4.2084
2009	0.0357	1	4	4	28.57	4.4341
2013	0.0362	1	5	5	35.71	4.6339
2011	0.0375	1	6	6	42.86	4.8200
2019	0.0376	1	7	7	50.00	5.0000
2017	0.0388	1	8	8	57.14	5.1800
2012	0.0390	1	9	9	64.29	5.3661
2018	0.0411	1	10	10	71.43	5.5659
2010	0.0416	1	11	11	78.57	5.7916
2021	0.0430	1	12	12	85.71	6.0676
2020	0.0439	1	13	13	92.86	6.4652
2022	0.0460	1	14	14	98.21_a_	7.1002

^a^corrected by (1-1/4 *n)*100%.

**Table 7:** Grading results of capacity evaluation of PHC service in China from 2009 to 2022.

Grade	*Probit*	Years
Poor	<4	2015, 2014
Medium	4 ~ 6	2016, 2009, 2013, 2011, 2019, 2017, 2012, 2018, 2010
Good	>6	2021, 2020, 2022

### Fuzzy comprehensive evaluation results

According to the fuzzy comprehensive evaluation of PHC service capacity in each year, the top 3 from 2009 to 2022 were in 2022, 2021 and 2020, and the last 3 were in 2015, 2014 and 2016. The results shows in Table 8.

**Table 8:** The fuzzy comprehensive evaluation of PHC service capacity in China from 2009 to 2022.

Years	TOPSIS		RSR		The fuzzy combination of TOPSIS and RSR						Comprehensive rank
	*C_i_*	Rank	*WRSR*	Rank	0.1*C*_i_ + 0.9W*RSR*	Rank	0.5*C*_i_ + 0.5W*RSR*	Rank	0.9*C*_i_ + 0.1W*RSR*	Rank	
2009	0.0740	7	0.0357	11	0.0396	9	0.0548	7	0.0701	7	7
2010	0.0634	8	0.0416	4	0.0438	7	0.0525	8	0.0612	8	8
2011	0.0536	13	0.0375	9	0.0391	10	0.0455	11	0.0520	12	11
2012	0.0599	9	0.0390	6	0.0411	8	0.0494	9	0.0578	9	9
2013	0.0572	10	0.0362	10	0.0383	11	0.0467	10	0.0551	10	10
2014	0.0537	12	0.0327	13	0.0348	13	0.0432	13	0.0516	13	13
2015	0.0526	14	0.0293	14	0.0316	14	0.0410	14	0.0503	14	14
2016	0.0551	11	0.0333	12	0.0354	12	0.0442	12	0.0529	11	12
2017	0.0930	6	0.0388	7	0.0442	6	0.0659	6	0.0876	6	6
2018	0.1015	5	0.0411	5	0.0471	5	0.0713	5	0.0955	5	5
2019	0.1349	4	0.0376	8	0.0473	4	0.0863	4	0.1252	4	4
2020	0.1527	3	0.0439	2	0.0548	3	0.0983	3	0.1418	3	3
2021	0.2626	2	0.0430	3	0.0650	2	0.1528	2	0.2407	2	2
2022	0.9299	1	0.0460	1	0.1344	1	0.4879	1	0.8415	1	1

### Sensitivity analysis

**Figure 1:**
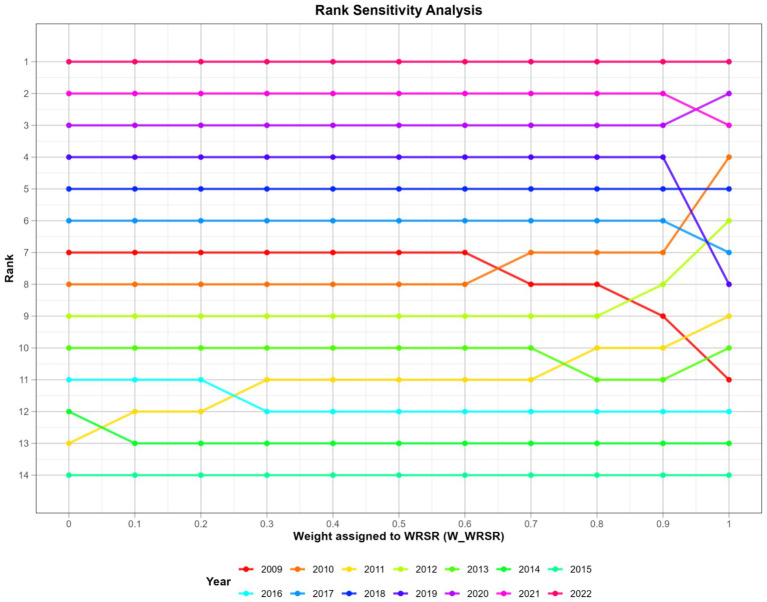
Sensitivity analysis of rank of fuzzy comprehensive evaluation.

Figure 1 sensitivity analysis intuitively reveals that the evaluation results change with the change of the weight of different analysis methods. The horizontal axis (X-axis) represents the weight assigned to the *WRSR* value. The vertical axis (Y-axis) represents the comprehensive ranking. Each unique colored line represents 1 year. By observing the line patterns, we can categorize these years into several categories:

1. Highly Stable Years (2022, 2021, 2018, 2016, 2015, 2014, 2013).

The lines for these years are almost completely horizontal. This indicates that regardless of whether the TOPSIS or RSR results are weighted, the final rankings remain largely unchanged.

1. Moderately Sensitive Years (2020, 2019, 2017).

The lines for these years are mostly flat, but within certain weight ranges, there are shifts of one or two rankings. The rankings for these years are relatively stable, but there are small fluctuations in weight ranges where the two methods diverge.

1. Highly Sensitive Years (2010, 2009, 2011, 2012).

The lines for these years intersect significantly on the graph. This indicates that the two evaluation methods have significantly different assessments of these years, and their final ranking is highly dependent on the choice of weights.

Overall, the comprehensive fuzzy evaluation is quite stable, with rankings remaining remarkably stable for most years (especially the best and worst performing years). While both TOPSIS and RSR are comprehensive evaluation methods, their underlying logic differs: TOPSIS is based on spatial distance, while RSR is based on ranking. This leads to discrepancies in the evaluation of certain years. The use of fuzzy comprehensive evaluation is particularly sensible, as it aims to smooth out the uncertainty introduced by weight selection and find the most robust comprehensive ranking across all possible scenarios.

## Discussion

This study utilized an entropy-weighted TOPSIS and RSR method comprehensive evaluation to provide a multi-dimensional, objective evaluation of China’s primary health care (PHC) service capacity from 2009 to 2022. Our findings reveal a complex picture: while the overall capacity shows a general upward trend, peaking in 2022, this growth is not uniform and is marked by significant fluctuations and internal contradictions across different dimensions. A pivotal finding of this study is the stark divergence between resource input and service efficiency. As shown in Table 4, the “Health Resource Input” dimension has steadily improved, with recent years like 2019, 2020, and 2018 ranking at the top. This reflects the success of China’s long-term policy of strengthening PHC infrastructure, evidenced by the rising numbers of health professionals (X2) and beds (X3) per 10,000 people. However, this investment has not translated into proportional gains in efficiency. Strikingly, “the Health Service Efficiency” dimension shows a declining trend, with the most efficient years being at the beginning of the study period (2009, 2010, 2011). This suggests a potential decoupling of investment and performance, where an increase in resources coincides with lower bed utilization rates (X8, X9) and potentially longer hospital stays (X10, X11). This phenomenon, where resource expansion outpaces efficiency gains, has been identified as a critical challenge in the next stage of China’s health system reform (6, 16–18).

We also observed that overall capacity rankings remained high in 2020 and 2021 despite a slight drop in routine services like treatments and hospitalizations. The entropy weighting method provides a data-driven explanation. Indicator X14 “Number of family health services” received the highest weight in the entire system, reflecting its significant and consistent growth over the study period. In contrast, indicators for routine patient visits (e.g., X5, X6, X7) received lower weights. This objectively demonstrates that the massive expansion of government-promoted family doctor contract services, a key policy priority, had a much larger impact on the comprehensive evaluation than the temporary, pandemic-induced decline in conventional patient interactions. This finding underscores a potential strategic shift in China’s PHC from a reactive, treatment-based model to a proactive, population-management-based model (19, 20).

Furthermore, the robustness of our overall findings is supported by the sensitivity analysis (Figure 1). While the rankings of early years (e.g., 2009–2012) were more sensitive to the weighting between TOPSIS and RSR, the rankings for most years, especially the top and bottom performers, remained remarkably stable. This confirms that the comprehensive evaluation is not an artifact of arbitrary methodological choices but reflects a consistent underlying trend, thus addressing a key concern about the validity of the results. The use of an objective weighting method combined with a comprehensive sensitivity analysis represents a significant methodological enhancement for studies in this field.

Placing these findings in a global context reveals the unique trajectory of China’s PHC development. While many developed nations, such as the United Kingdom, built their health systems upon general practice from the outset to control costs and ensure efficiency, China’s path has been different. The UK’s National Health Service has historically relied on patient registration with a specific general practitioner, who manages all aspects of care and acts as a single point of referral, a system proven to enhance care coordination and reduce unnecessary specialist visits (21, 22). China’s rapid, top-down, resource-heavy investment approach, while impressive in scale, has bypassed this foundational step, leading to the observed efficiency paradox. This situation also contrasts with many low- and middle-income countries, where the primary challenge is often absolute resource scarcity, forcing the adoption of innovative, efficiency-focused strategies like task-shifting from doctors to nurses or community health workers (23). China’s challenge is arguably more complex: it is not a lack of resources, but a systemic inefficiency in how those abundant resources are organized, financed, and utilized (24–27).

China’s burgeoning aging population and the evolving disease spectrum put forward higher requirements for primary health management (26). Drawing on these international experiences, several strategic recommendations can be proposed to address China’s PHC efficiency problem. First, strengthening the “gatekeeper” function of primary care is paramount. This requires moving beyond voluntary family doctor contracts toward more structured patient enrollment or registration systems, creating a stable doctor-patient relationship and a clear pathway for referrals (28–30). Second, leveraging digital health tools offers a powerful way to enhance efficiency. Integrating electronic health records, tele-health consultations, and remote monitoring can improve care coordination, support chronic disease management, and optimize the use of health professionals’ time, helping to bridge the gap between resource investment and performance output (31–33). Ultimately, the success of these systemic reforms depends on the quality and motivation of the primary care workforce itself. For the existing workforce, establishing a robust system of continuing professional development is essential to maintain and update clinical skills. To attract and retain high-caliber talent in primary care, general practitioner remuneration must be made competitive with that of hospital-based specialists, and a clear, rewarding career ladder must be established (34–36).

Nonetheless, our study has several limitations. Firstly, by using aggregated national data, it cannot capture the vast geographical and socio-economic disparities in PHC capacity between China’s eastern and western regions, or between urban and rural areas. Future research should urgently focus on provincial-level or stratified analyses to provide more targeted policy insights. Secondly, our indicator set, constrained by data availability from yearbooks, does not include crucial dimensions such as patient satisfaction, quality of care, or out-of-pocket expenses, which are vital for a truly holistic assessment. Finally, while our model identifies trends and correlations, it cannot establish causality. Deeper econometric or qualitative analyses are needed to explore the specific policy drivers behind the observed trends.

## Conclusion

In conclusion, this study demonstrates that while China’s primary health care service capacity has grown between 2009 and 2022, this development is primarily driven by a massive expansion in resources and a shift toward new service models like family doctor contracts. This growth, however, masks a persistent and worrying decline in service efficiency, indicating that simply increasing inputs is no longer a sufficient strategy. The objective weighting and dimensional analysis employed here reveal a critical need for a policy pivot from resource accumulation to performance optimization. Future efforts must focus on improving the operational efficiency of existing assets, enhancing the quality of care, and ensuring that investments translate into tangible and equitable health outcomes for the population.

## Data Availability

The original contributions presented in the study are included in the article/supplementary material, further inquiries can be directed to the corresponding author.
